# Supplementary material for: Design, optimization and analysis of large DNA and RNA nanostructures through interactive visualization, editing and molecular simulation
Source: Nucleic Acids Res. 2020 May 25;48(12):e72. doi: 10.1093/nar/gkaa417 (PMC7337935; doi:10.1093/nar/gkaa417)
Supplement: gkaa417_Supplemental_Files [file gkaa417_supplemental_files.zip › resubmission-supplementary.pdf]

Supplementary Material for "Design, optimization, and analysis of large DNA and RNA nanostructures through interactive visualization, editing, and molecular simulation"

Erik Poppleton,[1] Joakim Bohlin,[2] Michael Matthies,[1] Shuchi Sharma,[1] Fei Zhang,[3] and Petr Šulc[1]

[1] *Center for Molecular Design and Biomimetics at the Biodesign Institute and School of Molecular Sciences, Arizona State University, Tempe, Arizona 85287, United States*

[2] *Department of Physics, Clarendon Laboratory, University of Oxford, Parks Road, Oxford OX1 3PU, United Kingdom*

[3] *Department of Chemistry, Rutgers University-Newark, 73 Warren St, Newark, NJ 07102, USA*

SI. OXDNA INPUT AND OUTPUT FILES FORMAT

As the analyses discussed in the main text process the data from oxDNA files, it warrants a brief description of the file types encountered when running and analyzing oxDNA files. This will mitigate confusion as to why certain files are used in each case.

The three most important files are the input parameter file, the topology file and the trajectory file. The input file is a text file with the simulation's parameter names and values separated by equal signs. This file defines simulation and physical parameters such as temperature, the force field used, and file I/O information. Input files will be used by any analysis that calculates energy (primarily those concerned with hydrogen bonds) or is optimized by writing its most computationally-intensive operations distributed and compiled with the oxDNA code.

Topology files define the nucleotides present in the simulation. It is a text file with the extension .top and contains a header line with the number of nucleotides and the number of strands separated by a space. Following the header, each line defines a nucleotide with four values: the strand number, the base identity, 3' covalent connection, and 5' covalent connection (note that oxDNA numbers bases 3'-5', the reverse of the biochemistry convention). Strand ends have a value of -1.

The trajectory file contains the position, orientation and velocity of every nucleotide at each timepoint, based on the interval specified in the input file. Each configuration in the trajectory begins with a three-line header, defining the temperature, simulation box dimensions, and kinetic, potential, and total energies of the system. Each subsequent line defines one nucleotide with 15 parameters: position, orientation defined by two orthogonal vectors, translational velocity and rotational velocity. All parameters are in XYZ coordinates. The TacoxDNA webserver [1] has a variety of conversion tools from popular nanotechnology design tools and simulation formats into the oxDNA format. Once converted, these files can be visualized and edited using oxView or simulated using oxDNA/oxRNA. However, for large and/or long simulations, these files can become quite unwieldy, requiring tens of gigabytes of storage and therefore are impossible to open and read in a single reading frame. Therefore, all analyses and visualizations described in the main text read trajectory files in a stream, allowing reading of files that would not otherwise fit in the computer's RAM.

SII. SIMULATION DETAILS

The simulations given as examples here were run using the oxDNA code (June 2019 version). Structures were originally obtained in either Tiamat[2] or CaDNAno[3] format and then exported to oxDNA format using the TacoxDNA webserver [1]. Structures were relaxed in two steps. First, a brief Monte-Carlo (MC) simulation was performed using the DNA_relax or RNA_relax force fields to remedy overlapping particles. After this initial relaxation, mutual traps based on the intended design were applied to enforce relaxation to the intended design. For designs exported from Tiamat, mutual trap files are produced by TacoxDNA; for other structures, these files were produced using the force file generation script described in the "other utilities" section after the MC relax. A further relaxation was performed using the max_backbone_force option in a molecular dynamics (MD) simulation with the DNA2 or RNA2 force field. This bypasses checks of backbone bond length and allows for faster relaxation due to the CUDA implementation of the MD method in oxDNA [4]. This simulation was run until the energy stabilized between -57.98 and -62.13 pN nm (-1.4 and -1.5 oxDNA energy units respectively). At which point, the external forces and backbone force limitations were released and a production simulation run was performed using the same force field for 10^9 steps with a stepsize of 15.15 fs (0.005 oxDNA time units). This corresponds to a total run time in the microsecond range;

however, previous work with the oxDNA model [5, 6] suggests, in part, due to the increased diffusion coefficient, this direct conversion is an underestimate of the corresponding experimental time. However, as is the problem with all coarse-grained models, it is impossible to establish a direct correspondence between the simulation and experimental time because different processes in a coarse-grained simulation can scale to the experiment with different ratios.

The production simulations were performed at 20°C using an Andersen-like thermostat [7], and configurations were saved for analysis every 5×10^5 steps, resulting in 2000 separate configurations used in each analysis.

The RNA tile, used as an example of the clustering algorithm, was initially run as described above, however simulation runs would frequently transition to a state where one of the crossovers was broken. Further simulation only sampled the broken state and failed to capture reversible transition. Assessing the free-energy difference between states through simulation requires observing the transition multiple times. To facilitate observing the transition, a parallel tempering simulation using virtual-move Monte Carlo (VMMC) [8] with umbrella sampling was performed. Simulations with eight parallel replicas were performed with the temperatures set from 25 to 60°C at 5 degree increments. Replica exchange was attempted every 1000 steps and the average exchange acceptance rate was 0.47. Achieving reversible transitions along this order parameter also required creation of a weight file to bias the simulation towards transition states between the two states. This simulation successfully sampled multiple transitions between the two states and the resulting combined trajectory was used to demonstrate the clustering script.

SIII. RNA TILE ANALYSIS WITH UNSUPERVISED CLUSTERING

The trajectory files from all replicates of an RNA tile simulation discussed in the main text were combined into a single trajectory file containing 1345 configurations. The output from the principal component script was fed into the DBSCAN clustering algorithm as implemented in python sci-kit with parameters eps = 12 and min_samples = 8. This generated 5 different clusters of configurations, of which the first three are displayed in Fig. 11 of the main text. The number of configurations in each cluster were 812, 240, 110, 78 and 7 with 98 unclustered configurations. It is possible to have fewer configurations in a cluster than min_samples because min_samples only sets the number of neighbors required for a single point to be in a cluster. The last two clusters correspond to partially melted states stabilized by oxRNA's extremely strong cross-stacking interactions. We do not believe that these states are physically relevant, however they are successfully separated out by the clustering algorithm. The positions of each configuration projected to the first three axes in principal component space are shown in Fig. S1.

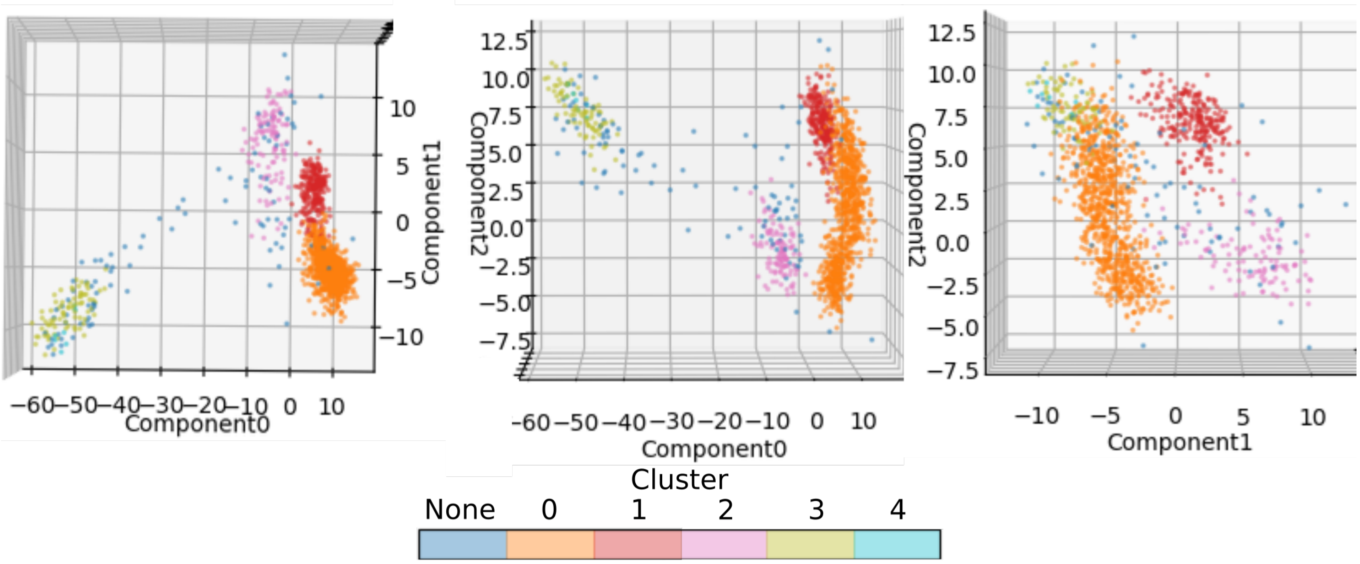

FIG. S1. The positions of each configuration projected to the first three dimensions of principal component space. The three plots correspond to looking down components 2, 1, and 0, respectively. Clusters 0, 1 and 2 correspond to the designed tile structure, the designed structure with stacking interrupted at the nick point, and the structure where the paranemic cohesion is lost becoming a Holliday junction. clusters 3 and 4 correspond to non-physically relevant states where the entire strand becomes a single hairpin.

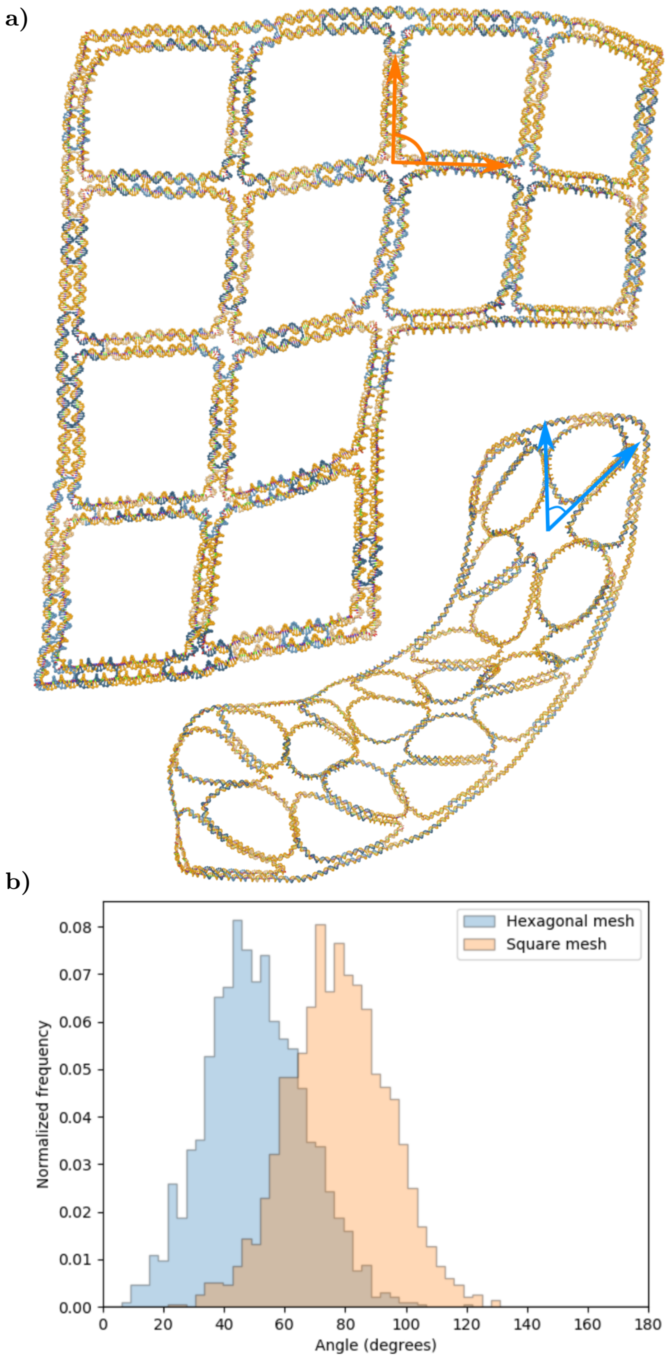

FIG. S2. **Comparing angles in wireframe lattices**. **a)** The mean structures of design 23 (top) and design 20 (bottom) from [9]. The structures are designed to have a square and hexagonal lattice pattern, respectively. **b)** The distribution of angles between two arms of a junction showing variation around the designed junction angle. For the hexagonal lattice, the observed angle is lower than the designed angle of 60° because the structure has significant out-of-plane curvature in the simulation.

SIV. INTERDUPLEX ANGLES AND DISTANCES

Figures S2 and S3 illustrate examples of analysis of interduplex angles and distances between origami units respectively, as discussed in the main text.

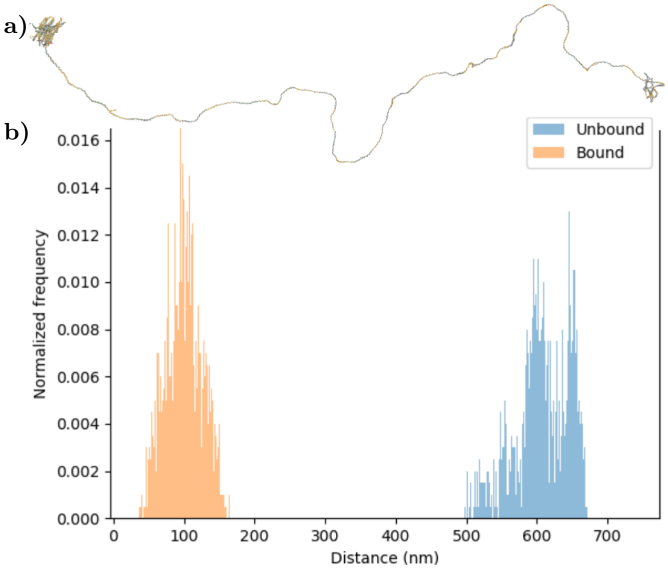

FIG. S3. **Distance between origami units of TMF**. **a)** The final configuration from a simulation of the TMF structure used in DNA kinetics experiments [10]. Separate simulations were performed with the sticky ends in both the bound and free configurations. **b)** The distribution of distances between the origami units at opposite ends of the tether.

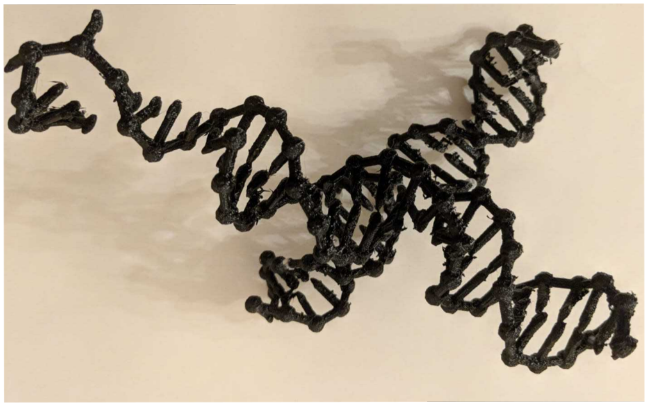

FIG. S4. **3D printed Holliday junction exported from oxView.** OxView supports export to GLTF format that can be opened in a 3D rendering tool Blender and exported to 3D printers or used for creation of more artistic 3D figures of DNA and RNA nanostructures.

---

[1] A. Suma, E. Poppleton, M. Matthies, P. Šulc, F. Romano, A. A. Louis, J. P. Doye, C. Micheletti, and L. Rovigatti. TacoxDNA: A user-friendly web server for simulations of complex DNA structures, from single strands to origami. *Journal of computational chemistry*, 40(29):2586–2595, 2019.

[2] S. Williams, K. Lund, C. Lin, P. Wonka, S. Lindsay, and H. Yan. Tiamat: A Three-Dimensional Editing Tool for Complex DNA Structures. In A. Goel, F. C. Simmel, and P. Sosík, editors, *DNA Computing*, pages 90–101, Berlin, Heidelberg, 2009. Springer Berlin Heidelberg.

[3] S. M. Douglas, A. H. Marblestone, S. Teerapittayanon, A. Vazquez, G. M. Church, and W. M. Shih. Rapid prototyping of 3D DNA-origami shapes with caDNAno. *Nucleic Acids Research*, 37(15):5001–5006, 2009.

[4] L. Rovigatti, P. Šulc, I. Z. Reguly, and F. Romano. A comparison between parallelization approaches in molecular dynamics simulations on GPUs. *Journal of computational chemistry*, 36(1):1–8, 2015.

[5] B. E. Snodin, F. Romano, L. Rovigatti, T. E. Ouldridge, A. A. Louis, and J. P. Doye. Direct simulation of the self-assembly of a small DNA origami. *ACS nano*, 10(2):1724–1737, 2016.

[6] M. C. Engel, D. M. Smith, M. A. Jobst, M. Sajfutdinow, T. Liedl, F. Romano, L. Rovigatti, A. A. Louis, and J. P. Doye. Force-induced unravelling of DNA origami. *ACS nano*, 12(7):6734–6747, 2018.

[7] J. Russo, P. Tartaglia, and F. Sciortino. Reversible gels of patchy particles: role of the valence. *The Journal of chemical physics*, 131(1):014504, 2009.

[8] S. Whitelam and P. L. Geissler. Avoiding unphysical kinetic traps in Monte Carlo simulations of strongly attractive particles. *Journal of Chemical Physics*, 127(15), 2007.

[9] H. Jun, F. Zhang, T. Shepherd, S. Ratanalert, X. Qi, H. Yan, and M. Bathe. Autonomously designed free-form 2D DNA origami. *Science advances*, 5(1):eaav0655, 2019.

[10] M. Schickinger, M. Zacharias, and H. Dietz. Tethered multifluorophore motion reveals equilibrium transition kinetics of single DNA double helices. *Proceedings of the National Academy of Sciences*, 115(39):201800585, 2018.
